# Validation of a General and Sports Nutrition Knowledge Questionnaire in Italian Early Adolescents

**DOI:** 10.3390/nu12103121

**Published:** 2020-10-13

**Authors:** Alice Rosi, Cinzia Ferraris, Monica Guglielmetti, Erika Meroni, Melanie Charron, Roberto Menta, Federica Manini, Vito Di Gioia, Daniela Martini, Daniela Erba

**Affiliations:** 1 Human Nutrition Unit, Department of Food and Drug, University of Parma, 43125 Parma, Italy; alice.rosi@unipr.it; 2 Human Nutrition and Eating Disorder Research Center, Department of Public Health, Experimental and Forensic Medicine, University of Pavia, 27100 Pavia, Italy; cinzia.ferraris@unipv.it (C.F.); monica.guglielmetti@unipv.it (M.G.); 3 Laboratory of Food Education and Sport Nutrition, Department of Public Health, Experimental and Forensic Medicine, University of Pavia, 27100 Pavia, Italy; 4 Department of Food, Environmental and Nutritional Sciences (DeFENS), Università degli Studi di Milano, 20122 Milan, Italy; erika.meroni@unimi.it; 5 Soremartec Italia Srl, Ferrero Group, 12051 Alba (CN), Italy; melanie.CHARRON@ferrero.com (M.C.); roberto.menta@ferrero.com (R.M.); federica.MANINI@ferrero.com (F.M.); 6 Federazione Italiana Giuoco Calcio, 00198 Rome, Italy; v.digioia@figc.it

**Keywords:** nutrition knowledge, sports nutrition, questionnaire, adolescents

## Abstract

To the best of our knowledge, no specific questionnaires on sports nutrition knowledge (NK) have been validated so far in Italian early adolescents. The aim of the present study was to validate a short (26-item) general and sports NK questionnaire in a group of Italian early adolescents. To this aim, the questionnaire was administered to 264 subjects for analysis of internal consistency, and in a subgroup (*n* = 39) for evaluating the reliability over time. The questionnaire revealed good overall internal consistency and reliability (Cronbach’s α = 0.684) and a highly significant correlation over time (r = 0.977, *p* < 0.001). Comparison with other validated questionnaires is tricky, because the previous questionnaires were validated in different populations, such as middle or late adolescents or adults, with a higher number of items compared to our questionnaire. Furthermore, data on adolescent NK in Italy are very limited. This study provides a brief, feasible, and validated questionnaire that can be used for investigating sports NK in young subjects. It could be used for evaluating the efficacy of education on general and sports nutrition in both the general population and athletes, and for investigating the relationship between NK and different sports in early adolescence.

## 1. Introduction

Nutrition knowledge (NK) is the ability to understand healthy nutrition concepts, and it may influence food behavior and eating habits that in turn can affect the health status [1]. This has led to a growing interest in NK, in such a way that several tools have been developed and validated in the recent years [2,3,4,5,6,7,8,9]. Most of them aimed to describe the NK level of a specific population in a qualitative–quantitative way, while others aimed to evaluate whether NK could influence dietary habits and food choices [10,11,12,13], as well as the effectiveness of nutrition education programs [14].

In the recent years, sports NK has also been investigated through specific questionnaires [15,16,17,18,19,20]. Sports nutrition can be defined as the application of NK to a nutritional plan focused on meeting the nutritional requirements for physical activity, optimizing the refueling process after physical exercise, and improving athletic performance in training and competitions, as well as promoting general health and well-being [21]. It has been pointed out that athletes with high confidence in their NK level felt able to apply this knowledge to their daily dietary behavior [22]. Similarly, coaches believe that athletes’ NK is one of the main factors influencing dietary practices [22].

In this context, several studies demonstrated inadequate NK and misunderstanding of general and sports-specific nutrition concepts, and this highlighted the need for nutrition education programs aimed at improving sports NK in athletes [13,23,24,25]. This seems to be particularly important in adolescents who are usually characterized by rapid physical, psychological, and emotional changes. Although many adolescent athletes believe that diet plays a key role in athletic performance, the specific nutritional needs for sports are not usually met [26,27,28]. Therefore, in order to optimize performance, health, and adequate development during adolescence, good eating habits and adequate intake of nutrients are essential, and an improved lifestyle may also be achieved through increased NK.

Taking this into account, many NK tools have been modified from previously validated instruments addressing general nutrition topics, with the aim of specifically validating questionnaires focused on sports NK [29,30,31,32,33]. Regarding tools focused on sports NK, questionnaires have been validated in different populations, including both athletes and coaches [15,25]. Recently, some new sports NK questionnaires for adult athletes have been developed, targeting track and field [16], ultra-endurance running [15,20], or other sports [17,18,34]. Most of the questionnaires were validated in adults [16,17,34], and just a few in adolescents [15,35].

Despite some NK questionnaires having been already validated in Italian adolescents [36,37], they focused on general NK. To the best of our knowledge, only one tool on sports NK has been validated in Italy on middle adolescents and young adults who practice sports at different levels [35]. This 62-item questionnaire is not easily used in younger populations such as early adolescents. In fact, the length may be a limiting factor for NK questionnaires validated in specific groups of population, especially for very young people or for athletes balancing training and study or who have many commitments [38]. In light of this evidence, data on Italian adolescent nutrition knowledge are very limited [35] and, to the best of our knowledge, no validated NK questionnaires are currently available to measure general and sports NK in Italian early adolescents.

Therefore, the aim of the present study was to develop and validate a short general and sports NK questionnaire in a group of Italian early adolescents, in the framework of a project on Nutrition Education and Sports signed by the Italian Society of Human Nutrition (SINU) in collaboration with the Federazione Italiana Giuoco Calcio (FIGC).

## 2. Materials and Methods

### 2.1. Development of the Questionnaire

Items of the questionnaire were collected by selecting pertinent items from existing general and sports NK questionnaires [34,35] or from previous research, in order to represent the entire topic under study. Initially, a pool of 100 items was generated by a group of eight nutritionists, including two experts in sports nutrition. According to previous studies, each expert on the panel was asked to indicate whether an item was “essential” or “not essential” to the operationalization of a theoretical construct. Then, the content validity ratio was calculated, yielding values that ranged from +1 to −1; positive values indicate that at least half of the experts rated the item as essential.

This process reduced the number of items to 26 in the final questionnaire, divided into 2 constructs. Construct 1 (General knowledge of nutrition) contained 16 questions focusing on the nutritional content of several foods, recommendation of nutrients, and the role of nutrients in health. Questions provided 3 options: True, False, or “I do not know.” Construct 2 (Sports nutrition) was composed of 10 items on the relationship between nutrition and sports (e.g., pretraining snack, water consumption during activities, and the use of supplements). Six questions provided 3 options: True, False, or “I do not know,” while the other 4 questions were multiple-choice with three to four options with only one correct choice and an “I do not know” option. These multiple-choice questions were considered particularly important to assess sports nutrition knowledge relating to an athlete’s dietary plan, so allowing 4 options increased the difficulty. Irrespective of the number of options, +1 point was assigned to a correct answer, and 0 points to the incorrect or “I do not know” options.

### 2.2. Administration and Validation of the Questionnaire

The questionnaire was administered to all participants under the same conditions and participants were asked to answer all questions. In a subgroup of the total population, the questionnaire was administered twice, separated by 3 weeks. The time interval between questionnaires was considered short enough to avoid real changes in nutrition knowledge, but long enough to avoid recall bias [39].

On the basis of the answers, a NK score was calculated for each section and for the overall questionnaire, giving 1 point for each correct answer and adding up the points from each question (Section 1 score range: 0–16; Section 2 score range: 0–10; and total questionnaire score range: 0–26).

Answers from the first questionnaire were used to perform an item analysis (item difficulty and item discrimination index) and to test the questionnaire’s internal consistency and construct validity, while test‒retest reliability was assessed using data from both assessments.

### 2.3. Subjects

Subjects attending the first grade of secondary school in Buccinasco (Italy) or the Federal Regional Centers (CFT) of the Italian Football Federation (FIGC), located in Verano Brianza, Corticella, and Casalnuovo, were invited to participate in this study. Participants were eligible if they were males or females aged between 12 and 14 years, with the exception of subjects on particular dietary regimes (e.g., celiac) and pregnant or breastfeeding adolescents. One parent of each participant gave his/her informed written consent to participate in the study. Parents and their children were assured of complete anonymity. Enrolment was performed during spring 2019.

The Ethics Committee of the Università degli Studi di Milano approved the study protocol (Protocol number 54/18).

### 2.4. Statistical Analysis

The sample size calculation was based on the “rule of thumb” (*n*:p) [40]. According to this rule, the ratio between the number of subjects (*n*) and the number of items of the questionnaire (p) should be 10. Since 26 items compose the questionnaire, at least 260 subjects should be enrolled in the study.

Single items were analyzed by means of the item difficulty index (percentages of correct answers for each individual question) and of the item discrimination index (point-biserial correlation between the score for each question and the total score). Despite the item difficulty index cutoffs often being set between 0.2 and 0.8 [41], we applied a conservative approach, fixing the cutoff between 0.1 and 0.9 [15,42,43]: values below 0.1 (10% or right responders) indicate that the questions are too difficult, while values above 0.9 (90% of right respondents) mean that the questions are too easy. For the item discrimination, the minimum item‒total correlation is usually set to 0.2 [41], with higher values corresponding to stronger correlations. Internal consistency was measured for the total questionnaire and for each construct (“General knowledge of nutrition” and “Sports nutrition”) by computing the Cronbach’s alpha. Even if the minimum requirement for internal consistency is often recommended to be 0.7 [42] for the classical test theory principles, several authors reported that the Cronbach’s alpha value is acceptable if over 0.6 [44,45,46]. In addition, a Pearson’s correlation was used as a measure of temporal stability (test‒retest reliability) for the total score, the score of each construct, and each individual item. Construct validity was tested by using a two-sample Student’s *t*-test, searching for differences in the total and partial NK scores between participants who did not receive nutrition lessons and participants who had nutrition lessons before the first administration of the questionnaire (T0).

The statistical analyses were performed using the Statistical Package for Social Science (IBM SPSS Statistics for Macintosh, version 26.0, IBM Corp., Armonk, NY, USA) and the significance was set to *p* < 0.05.

## 3. Results

### 3.1. Participants

A total of 366 adolescents were invited to take part in this study, and all subjects gave their consent to participate (total response rate: 100%). However, only adolescents who were present on the day of the assessment took part in the study (75%). Out of the total respondents, nine participants did not complete the questionnaire and were excluded; thus, a total of 264 subjects (age 12.6 ± 0.6 years, 71% males) were included in the final analysis (total completion rate: 97%). Among respondent subjects, 57% had never received any nutrition lessons, while 43% underwent nutrition education activities before the questionnaire. In addition, 67% of participants were football players involved in the activities at one of the Federal Regional Centers (CFT) of the Italian Football Federation (FIGC), who had 2–6 training sections each week plus a competition during the weekend. The other 33% of participants were enrolled in a school setting and were not semiprofessional athletes. Subjects enrolled at school were invited to complete the questionnaire twice for the test‒retest analysis; however, only 39 students were present at school during both assessment days (47% of participants enrolled at school and 14% of the total sample).

### 3.2. Item Analysis

Item difficulty and discrimination indexes for each question are shown in Table 1.

The rate of correct answers was lower than 0.2, but higher than 0.1 for two questions, both in the “Sports nutrition” section, while only two items had a difficulty index of 0.8–0.9 (one in each questionnaire section) and none of the questions were correctly answered by all the participants. Thus, all 26 items were kept in the final version of the questionnaire, since the exclusion of one or more items would not have improved the questionnaire reliability.

A poor (cutoff <0.2) [41,42] score correlation between the score on each question and the total score was observed for two items (both in the “Sports nutrition” section), while four items had values only slightly lower than 0.2. However, questions that scored below 0.2 were considered extremely important by the expert panel and kept in the final version of the questionnaire.

### 3.3. Questionnaire Reliability

A medium–low score was registered for the total questionnaire and for both sections (Table 2). The reliability was acceptable for the total questionnaire (Cronbach’s α = 0.684). When individual constructs were analyzed, a marginal internal consistency was achieved for construct 1 (General knowledge in nutrition), while the second construct (Sports nutrition) revealed a weak internal consistency.

An analysis of the reliability over time was carried out on a subgroup of the total population composed of 39 participants (age 13.1 ± 0.6 years) (Table 3). A significant correlation was found for both total score and individual construct scores (*r* > 0.9, *p* < 0.001, for all), while a medium value of the identical response to the same question was observed (48% for the total questionnaire and 50% and 46%, respectively, for construct 1 and construct 2).

### 3.4. Questionnaire Validity

When subjects expected to have different NK were compared (Table 4), a significantly higher total NK score (*p* < 0.001) was observed in participants who had some nutrition lessons compared to those who did not have nutrition lessons before the baseline assessment. Similarly, higher NK scores were obtained for the two questionnaire sections (General knowledge of nutrition: *p* = 0.003, Sports nutrition: *p* = 0.001).

## 4. Discussion

The aim of the present study was to assess the validity and reliability of a self-administered general and sports NK questionnaire administered to 264 early adolescents.

Out of the 100 items initially generated by the group of experts, 26 questions were included in the final version of the questionnaire. As regards the difficulty index cutoff, 0.1–0.9 was chosen, as in some of the other nutrition knowledge questionnaires targeted at young respondents [15,47,48]—items that were too easy or too difficult are generally removed from final questionnaires, although, in many studies on sports nutrition, this step is not reported [49,50,51,52]. The final questionnaire also included items with a low discrimination index, because they were considered extremely important to the topic. For instance, the first two questions of the sports nutrition section (“What is the best meal for an athlete increasing muscle mass?” and “Can vegetarian athletes satisfy their protein needs without protein supplementation?”) were considered important topics in sports nutrition, especially in adolescent athletes [49,53,54,55,56].

The questionnaire revealed an acceptable internal consistency reliability, in accordance with the Cronbach’s scores. Cutoff values for questionnaire reliability very often depend on the field of application, and generalization is not recommended. Despite the minimum internal consistency often being recommended as 0.7 [42,43], some authors reported that a Cronbach’s alpha value is acceptable if it is over 0.6 [44,45,46]. Accordingly, several papers presented questionnaire validation with a Cronbach’s alpha value ranging between 0.6 and 0.7 [57,58], as in our case.

In the present study, a total of 264 adolescents were included, 57% of whom had never received any nutrition information, while 43% had enrolled in a nutrition education program before the questionnaire administration. A significantly higher total NK score was observed in participants who had had some nutrition lessons compared to those who did not have nutrition information before the baseline assessment. These differences in knowledge score were statistically significant for the total questionnaire, but also for both the “General knowledge of nutrition” and “Sports nutrition” sections, thus demonstrating that our questionnaire can discriminate between adolescents with different knowledge levels. Similar to the present findings, in previous studies, people with potential differences in NK (e.g., dietitians versus other workers, students with or without a nutrition background) also showed higher NK compared to non-experts [4,16]. Other studies on sports nutrition evaluated possible differences between groups that received or did not receive nutritional education [15,16,35,38,50] but, in the majority of them, the participants were adults (i.e., college students or athletes) and not adolescents.

Comparison with previous questionnaires is tricky because of the differences between present and previously validated tools, for instance, in terms of the number of items, type of questions, and target populations. Among the studies validating questionnaires on general and sports NK, Furber and co-workers developed a 62-item questionnaire in a cohort of UK-based track and field athletes [16]. The authors compared NK between subjects with nutrition training (i.e., sports nutritionists and dietitians) and those without nutrition training (i.e., professionals and postgraduate students with no exposure to any form of nutrition training).

Other sports NK questionnaires were developed by Trakman and colleagues. In detail, after the validation of an 89-item sports NK questionnaire [38], a brief version (37 items) [34] was validated in 181 adult athletes, who were predominately female (61%) and aged 17–25 years (48%). In the same year, Heikkila and co-workers [15] validated a 79-item questionnaire for assessing the NK of young endurance athletes (*n* = 16, aged 15‒20 years) and their coaches (*n* = 13, aged 23‒52 years). Finally, general and sports NK has recently been correlated with better dietary intake in Brazilian adolescent (*n* = 73, aged 14‒19 years) soccer players [32], using a questionnaire previously validated in 19 graduates of nutrition programs and 16 adolescent athletes [59].

These previous studies were conducted on a target population with older subjects compared to our group. In addition, the number of items was often greater than ours, which may limit the use of the questionnaire by early adolescents. Moreover, the previously cited studies usually investigated general or sports NK, but none combined these two fields.

In Italy, Calella and colleagues [35] validated a questionnaire on general and sports NK in middle and late adolescents (i.e., 14‒19 years) and young adults. Thus, the main differences compared to our questionnaire were related both to the target population (early vs. middle and late adolescents) and to the >2-fold higher number of items (26 vs. 62 items). Other questionnaires were instead validated in Italian young people on different topics. Among those, Grosso and colleagues validated a questionnaire focusing on food nutrients, food contents, healthy food and drinks, and energy expenditure [36]. The authors obtained good internal consistency, except in the section related to lifestyle, and temporal stability. Another group of Italian researchers, instead, validated a 31-item questionnaire on several nutrition-related topics, finding poor knowledge about several topics including healthy diet, snacks, and milk and dairy products [37].

The present study has some strengths and limitations worth highlighting. First, to the best of our knowledge, this study is the first one providing a validated questionnaire to be utilized in early adolescents practicing sports or not, focusing on both general and sports NK. Moreover, the limited number of items makes this questionnaire more suitable for achieving higher completion rates (97%). In fact, our completion rate is higher than that calculated by Trackman (66%) during the validation of the 89-item questionnaire [38]. Therefore, this underlines how a shorter questionnaire can be more practical in certain specific environments such as a sports centers or classroom. In fact, it is likely that long questionnaires would be discouraging to athletes and their coaches, and this may at least partially explain the low response and completion rates of previous general and sports NK questionnaire [34]. A limitation to be highlighted is related to the limited samples used for the test‒retest evaluation. Despite this, the ratio between subjects enrolled for measuring the internal consistency and the temporal stability (264 and 39, respectively, thus ~15%) is in line with that used in previous investigations in both sports nutrition [38] and in other types of questionnaires [6,60]. Another limitation to note is that the assessment of the criterion validity was outside of the scope of this study, and should be addressed in future research to ensure complete validation of the new survey. Last, it should be highlighted that the Cronbach’s alpha for internal consistency was 0.684, slightly lower than the 0.7 cutoff considered more appropriate by some authors [42,44,45,46]. Nevertheless, despite the intense debate about this topic, this cutoff should be considered as a guide rather than absolute [61], as it depends on several factors related to the complexity of the questionnaire. In this sense, the borderline Cronbach’s alpha reported herein provided an indication of the adequate reliability of the questionnaire.

## 5. Conclusions

In conclusion, this study provides a brief, feasible, and validated questionnaire that lends itself to use for investigating NK, even in young subjects. As reported by Tam and co-workers, only 15.6% of education programs designed to improve nutrition knowledge in athletes use well-validated knowledge assessment tools [62]. Therefore, our questionnaire might be a useful instrument for evaluating the efficacy of nutrition education programs in both the general population and athletes.

Involving active adolescents while practicing sports provides a ‘window of opportunity’ to cultivate life skills that support wellness and obesity prevention, such as healthy eating behaviors and meal planning. This is also an opportunity to teach young athletes how to have adequate hydration and energy supply for sports and health as well as to discern whether sports supplements are needed [63].

Moreover, this questionnaire could be used in future research studies in order to investigate the relationship between nutritional knowledge and sports activities, as well as differences in nutritional knowledge for different sports and sport categories in early adolescents.

## Figures and Tables

**Table 1:** Item analysis for the 26 questions of the questionnaire.

Item	Item Difficulty	Item Discrimination
	(Correct Answers)	(*r* Value)
*General knowledge in nutrition*		
1—protein intake and body fat	62%	0.307
2—carbohydrate content of banana	30%	0.198
3—carbohydrate content of rice	56%	0.249
4—fat requirements	72%	0.197
5—fat content of low-fat cheese	50%	0.363
6—fat content of butter	89%	0.391
7—fat content of honey	61%	0.319
8—daily recommended intake of water	76%	0.372
9—protein content of hard cheese	56%	0.259
10—protein content of beans	53%	0.325
11—protein content of corn	43%	0.317
12—protein quality of eggs	62%	0.365
13—energy from vitamins	23%	0.216
14—daily recommended calcium intake	22%	0.223
15—alcohol and body fat	58%	0.279
16—alcohol intake and recovery from injuries	54%	0.376
*Sports nutrition*		
17—the best meal for increasing muscle mass	41%	0.194
18—protein needs of vegetarian athletes	46%	0.205
19—vitamin and mineral supplements and sport performance	25%	0.268
20—water consumption during training	12%	0.142
21—dehydration and sport performance	84%	0.391
22—snacking during training	61%	0.310
23—carbohydrate intake during training	41%	0.161
24—label and claims on food supplements	39%	0.338
25—safety of supplements	13%	0.190
26—doping substances	39%	0.244

The two main constructs of the questionnaire are in Italic.

**Table 2:** Internal consistency.

Section	Score Range	Baseline NK Score	Cronbach’s Alpha
		(Mean ± SD)	
1—General knowledge of nutrition	0‒16	8.7 ± 2.5	0.574
2—Sports nutrition	0‒10	4.0 ± 1.5	0.390
Total questionnaire	0‒26	12.7 ± 3.3	0.684

NK: nutrition knowledge.

**Table 3:** Test–retest reliability.

Section (*n* of Items)	Baseline NK Score (T0)	Follow-up NK Score (T1)	Correlation		Identical Responses Rates
			(Total Scores)		
	Mean ± SD	Mean ± SD	R	*p*-Value *	%
1—General knowledge of nutrition	8.6 ± 1.6	8.7 ± 1.4	0.975	<0.001	49.7
2—Sports nutrition	3.9 ± 1.5	3.5 ± 1.3	0.937	<0.001	46.4
Total questionnaire	12.5 ± 2.3	12.3 ± 2.4	0.977	<0.001	48.4

* Pearson’s correlation test with significance of *p* < 0.05.

**Table 4:** Construct validity.

Section	NK score Non-Nutrition Lesson Group (*n* = 151)	NK Score Nutrition Lesson Group (*n* = 113)	*p*-Value
	(Mean ± SD)	(Mean ± SD)	
1—General knowledge of nutrition	8.3 ± 2.3	9.2 ± 2.6	0.003
2—Sports nutrition	3.7 ± 1.5	4.4 ± 1.5	0.001
Total questionnaire	12.0 ± 3.0	13.6 ± 3.4	<0.001

## References

[1] Worsley A. (2002). Nutrition knowledge and food consumption: Can nutrition knowledge change food behaviour?. Asia Pac. J. Clin. Nutr..

[2] Alkaed D., Ibrahim N., Ismail F., Barake R. (2018). Validity and Reliability of a Nutrition Knowledge Questionnaire in an Adult Student Population. J. Nutr. Educ. Behav..

[3] Alsaffar A.A. (2012). Validation of a general nutrition knowledge questionnaire in a Turkish student sample. Public Health Nutr..

[4] Rosi A., Martini D., Grosso G., Bonaccio M.L., Scazzina F., Angelino D. (2020). Validation of a nutrition knowledge questionnaire in Italian students attending the University of Parma. Public Health Nutr..

[5] Kliemann N., Wardle J., Johnson F., Croker H. (2016). Reliability and validity of a revised version of the General Nutrition Knowledge Questionnaire. Eur. J. Clin. Nutr..

[6] Sachdev P.K., Freeland-Graves J., Babaei M. (2020). Development and validation of the Dental Nutrition Knowledge Competency Scale for low-income women. Public Health Nutr..

[7] Rovner A.J., Nansel T.R., Mehta S.N., Higgins L.A., Haynie D.L., Laffel L.M. (2012). Development and Validation of the Type 1 Diabetes Nutrition Knowledge Survey. Diabetes Care.

[8] Iaccarino Idelson P., D’Elia L., Cairella G., Sabino P., Scalfi L., Fabbri A., Galletti F., Garbagnati F., Lionetti L., Paolella G. (2020). Salt and Health: Survey on Knowledge and Salt Intake Related Behaviour in Italy. Nutrients.

[9] Deniz M.S., Alsaffar A.A. (2014). Assessing the Validity and Reliability of a Questionnaire on Dietary Fibre-related Knowledge in a Turkish Student Population. J. Health Popul. Nutr..

[10] Argôlo D., Borges J., Cavalcante A., Silva G., Maia S., Moraes A., Oliveira E., Nascimento M. (2018). Poor dietary intake and low nutritional knowledge in adolescent and adult competitive athletes: A warning to table tennis players. Nutr. Hosp..

[11] Yahia N., Brown C.A., Rapley M., Chung M. (2016). Level of nutrition knowledge and its association with fat consumption among college students. BMC Public Health.

[12] Hardy R., Kliemann N., Evansen T., Brand J. (2017). Relationship Between Energy Drink Consumption and Nutrition Knowledge in Student-Athletes. J. Nutr. Educ. Behav..

[13] Spronk I., Heaney S.E., Prvan T., O’Connor H.T. (2015). Relationship Between General Nutrition Knowledge and Dietary Quality in Elite Athletes. Int. J. Sport Nutr. Exerc. Metab..

[14] Kourlaba G., Panagiotakos D.B. (2009). Dietary quality indices and human health: A review. Maturitas.

[15] Heikkilä M., Valve R., Lehtovirta M., Fogelholm M. (2018). Development of a nutrition knowledge questionnaire for young endurance athletes and their coaches. Scand. J. Med. Sci. Sports.

[16] Furber M.J.W., Roberts J.D., Roberts M.G. (2017). A valid and reliable nutrition knowledge questionnaire for track and field athletes. BMC Nutr..

[17] Andrews A., Wojcik J.R., Boyd J.M., Bowers C.J. (2016). Sports Nutrition Knowledge among Mid-Major Division I University Student-Athletes. J. Nutr. Metab..

[18] Karpinski C.A., Dolins K.R., Bachman J. (2019). Development and Validation of a 49-Item Sports Nutrition Knowledge Instrument (49-SNKI) for Adult Athletes. Top. Clin. Nutr..

[19] Tam R., Beck K.L., Gifford J.A., Flood V.M., O’Connor H.T. (2020). Development of an Electronic Questionnaire to Assess Sports Nutrition Knowledge in Athletes. J. Am. Coll. Nutr..

[20] Blennerhassett C., McNaughton L.R., Cronin L., Sparks S.A. (2019). Development and implementation of a nutrition knowledge questionnaire for ultraendurance athletes. Int. J. Sport Nutr. Exerc. Metab..

[21] Hedrick F.H., Mikesky A.E. (2018). Practical Applications in Sports Nutrition.

[22] Heaney S., O’Connor H., Michael S., Gifford J., Naughton G. (2011). Nutrition Knowledge in Athletes: A Systematic Review. Int. J. Sport Nutr. Exerc. Metab..

[23] Trakman G.L., Forsyth A., Devlin B.L., Belski R. (2016). A systematic review of athletes’ and coaches’ nutrition knowledge and reflections on the quality of current nutrition knowledge measures. Nutrients.

[24] Alaunyte I., Perry J.L., Aubrey T. (2015). Nutritional knowledge and eating habits of professional rugby league players: Does knowledge translate into practice?. J. Int. Soc. Sports Nutr..

[25] Spendlove J.K., Heaney S.E., Gifford J.A., Prvan T., Denyer G.S., O’Connor H.T. (2012). Evaluation of general nutrition knowledge in elite Australian athletes. Br. J. Nutr..

[26] Ferraris C., Guglielmetti M., Trentani C., Tagliabue A. (2019). Assessment of Dietary Under-Reporting in Italian College Team Sport Athletes. Nutrients.

[27] Manore M., Patton-Lopez M., Meng Y., Wong S. (2017). Sport Nutrition Knowledge, Behaviors and Beliefs of High School Soccer Players. Nutrients.

[28] Hassapidou M.N., Valasiadou V., Tzioumakis L., Vrantza P. (2002). Nutrient intake and anthropometric characteristics of adolescent Greek swimmers. Nutr. Diet..

[29] Devlin B.L., Belski R. (2015). Exploring General and Sports Nutrition and Food Knowledge in Elite Male Australian Athletes. Int. J. Sport Nutr. Exerc. Metab..

[30] Cupisti A., D’Alessandro C., Castrogiovanni S., Barale A., Morelli E. (2002). Nutrition knowledge and dietary composition in Italian adolescent female athletes and non-athletes. Int. J. Sport Nutr..

[31] Rosenbloom C.A., Jonnalagadda S.S., Skinner R. (2002). Nutrition knowledge of collegiate athletes in a Division I National Collegiate Athletic Association institution. J. Am. Diet. Assoc..

[32] Noronha D.C., Santos M.I.A.F., Santos A.A., Corrente L.G.A., Fernandes R.K.N., Barreto A.C.A., Santos R.G.J., Santos R.S., Gomes L.P.S., Nascimento M.V.S. (2020). Nutrition Knowledge is Correlated with a Better Dietary Intake in Adolescent Soccer Players: A Cross-Sectional Study. J. Nutr. Metab..

[33] Jessri M.M., Jessri M.M., RashidKhani B., Zinn C. (2010). Evaluation of Iranian College Athletes’ Sport Nutrition Knowledge. Int. J. Sport Nutr. Exerc. Metab..

[34] Trakman G.L., Forsyth A., Hoye R., Belski R. (2018). Development and validation of a brief general and sports nutrition knowledge questionnaire and assessment of athletes’ nutrition knowledge. J. Int. Soc. Sports Nutr..

[35] Calella P., Iacullo V., Valerio G. (2017). Validation of a General and Sport Nutrition Knowledge Questionnaire in Adolescents and Young Adults: GeSNK. Nutrients.

[36] Grosso G., Mistretta A., Turconi G., Cena H., Roggi C., Galvano F. (2013). Nutrition knowledge and other determinants of food intake and lifestyle habits in children and young adolescents living in a rural area of Sicily, South Italy. Public Health Nutr..

[37] Tallarini A., Zabeo A., Ferraretto A. (2014). Nutritional knowledge in an Italian population of children, pre-adolescents and adolescents. Public Health Nutr..

[38] Trakman G.L., Forsyth A., Hoye R., Belski R. (2017). The nutrition for sport knowledge questionnaire (NSKQ): Development and validation using classical test theory and Rasch analysis. J. Int. Soc. Sports Nutr..

[39] Feren A., Torheim L., Lillegaard I.L. (2011). Development of a nutrition knowledge questionnaire for obese adults. Food Nutr. Res..

[40] Everitt B.S. (1975). Multivariate Analysis: The Need for Data, and other Problems. Br. J. Psychiatry.

[41] Kline P. (2013). Handbook of Psychological Testing.

[42] Parmenter K., Wardle J. (2000). Evaluation and Design of Nutrition Knowledge Measures. J. Nutr. Educ..

[43] Trakman G.L., Forsyth A., Hoye R., Belski R. (2017). Developing and validating a nutrition knowledge questionnaire: Key methods and considerations. Public Health Nutr..

[44] Cronbach L.J. (1951). Coefficient alpha and the internal structure of tests. Psychometrika.

[45] De Vellis R.F. (2003). Scale Development: Theory and Applications (Applied Social Research Methods).

[46] Taber K.S. (2018). The Use of Cronbach’s Alpha When Developing and Reporting Research Instruments in Science Education. Res. Sci. Educ..

[47] Whati L.H., Senekal M., Steyn N.P., Nel J.H., Lombard C., Norris S. (2005). Development of a reliable and valid nutritional knowledge questionnaire for urban South African adolescents. Nutrition.

[48] Dwyer J.T., Stolurow K.A., Orr R. (1981). A nutrition knowledge test for high school students. J. Nutr. Educ..

[49] Barr S.I. (1987). Nutrition knowledge of female varsity athletes and university students. J. Am. Diet. Assoc..

[50] Zinn C., Schofield G., Wall C. (2005). Development of a psychometrically valid and reliable sports nutrition knowledge questionnaire. J. Sci. Med. Sport.

[51] Hoogenboom B.J., Morris J., Morris C., Schaefer K. (2009). Nutritional knowledge and eating behaviors of female, collegiate swimmers. N. Am. J. Sports Phys. Ther..

[52] Shoaf L.R., McClellan P.D., Birskovich K.A. (1986). Nutrition knowledge, interests, and information sources of male athletes. J. Nutr. Educ..

[53] Mazzulla M., Volterman K.A., Packer J.E., Wooding D.J., Brooks J.C., Kato H., Moore D.R. (2018). Whole-body net protein balance plateaus in response to increasing protein intakes during post-exercise recovery in adults and adolescents. Nutr. Metab..

[54] Bettonviel A.E.O., Brinkmans N.Y.J., Russcher K., Wardenaar F.C., Witard O.C. (2016). Nutritional Status and Daytime Pattern of Protein Intake on Match, Post-Match, Rest and Training Days in Senior Professional and Youth Elite Soccer Players. Int. J. Sport Nutr. Exerc. Metab..

[55] Borrione P., Spaccamiglio A., Salvo R.A., Mastrone A., Fagnani F., Pigozzi F. (2009). Rhabdomyolysis in a Young Vegetarian Athlete. Am. J. Phys. Med. Rehabil..

[56] Melina V., Craig W., Levin S. (2016). Position of the Academy of Nutrition and Dietetics: Vegetarian Diets. J. Acad. Nutr. Diet..

[57] Nahar B., Hossain M., Ickes S.B., Naila N.N., Mahfuz M., Hossain D., Denno D.M., Walson J., Ahmed T. (2019). Development and validation of a tool to assess appetite of children in low income settings. Appetite.

[58] Nackers F., Roederer T., Marquer C., Ashaba S., Maling S., Mwanga-Amumpaire J., Muny S., Sokeo C., Shom V., Palha M. (2019). A screening tool for psychological difficulties in children aged 6 to 36 months: Cross-cultural validation in Kenya, Cambodia and Uganda. BMC Pediatr..

[59] Rodrigues Leite M.D.M., Santos Barbosa Machado A.C., Da Silva D.G., Falcão Raposo O.F., Mendes Netto R.S. (2016). Conocimiento sobre alimentación y nutrición después del desarrollo de actividades de educación alimentaria entre niños y adolescentes deportistas. Pensar a Prática.

[60] Sasanfar B., Toorang F., Nemati S., Djazayery A., Zendehdel K. (2019). Development and validation of a knowledge, attitude, and practice questionnaire on nutrition-related cancer prevention for Iranian women. J. Res. Med. Sci..

[61] Lance C.E., Butts M.M., Michels L.C. (2006). The Sources of Four Commonly Reported Cutoff Criteria. Organ. Res. Methods.

[62] Tam R., Beck K.L., Manore M.M., Gifford J., Flood V.M., O’Connor H. (2019). Effectiveness of Education Interventions Designed to Improve Nutrition Knowledge in Athletes: A Systematic Review. Sport. Med..

[63] Patton-Lopez M., Manore M., Branscum A., Meng Y., Wong S. (2018). Changes in Sport Nutrition Knowledge, Attitudes/Beliefs and Behaviors Following a Two-Year Sport Nutrition Education and Life-Skills Intervention among High School Soccer Players. Nutrients.

